# Surgical Nurses’ Perceptions of Strategies to Enhance Pain Management Proficiency: A Qualitative Study

**DOI:** 10.3390/nursrep13020081

**Published:** 2023-06-15

**Authors:** Jarutsri Atthayasai, Manaporn Chatchumni, Henrik Eriksson, Monir Mazaheri

**Affiliations:** 1 School of Nursing, Rangsit University, Pathumthani 12000, Thailand; jarutsri.a@rsu.ac.th; 2 Department of Health Sciences, University West, 46132 Trollhättan, Sweden; henrik.eriksson@hv.se; 3 Department of Nursing Sciences, Sophiahemmet University, 11486 Stockholm, Sweden; monir.mazaheri@shh.se; 4 Department of Neurobiology, Care Sciences and Society, Karolinska Institutet, 14152 Stockholm, Sweden

**Keywords:** competence, knowledge, postoperative pain, pain management proficiency, surgical nurses’ strategies

## Abstract

To describe surgical nurses’ strategies for enhancing their pain management proficiency. A qualitative design was used to conduct the study. The participants were forty surgical nurses who had at least six years of nursing experience in caring for patients with pain. They responded to open-ended questions based on a review of the policy documents concerning the main elements of the pain management programme to be implemented by surgical nurses. Three key themes emerged from the surgical nurses’ suggested strategies: partnering, disrupting, and becoming familiar with pain management competency concerns. Surgical nurses’ strategies in acute and chronic pain management nursing units included solving patients’ problems and promoting and enhancing pain strategies to address health challenges in organisations. The themes presented in the results include enhancing pain management in nursing competencies. State-of-the-art healthcare technologies are being applied to pain management. Surgical nurses’ strategies should improve the quality of nursing care, especially post-surgery recovery time. It is recommended to engage patients, their families, and multidisciplinary care teams in other healthcare fields.

## 1. Introduction

Postoperative pain is an important issue that affects millions of individuals worldwide. It is a common outcome of surgical interventions and can have a profound impact on the quality of life, recovery, and overall well-being of patients [1,2]. The experience of post-operative pain can vary widely depending on factors such as the type and extent of surgery, individual pain tolerance, and the overall health of the patient; however, it is well-documented that inadequate pain control after surgery can lead to various negative short- and long-term outcomes [3,4]. The nursing strategies for surgical nurses are highly relevant to pain management practices that provide knowledge regarding today’s important health challenges [3,4]. Previous studies have indicated that nurses can improve their pain management skills through critical thinking, leadership, patient management, and health promotion. Samarkandi [4], and Jaleta, Tuji, and Wake [5] have focused on pain management related to ineffective nursing strategies. Inefficiencies exist in multidisciplinary collaborations, the development of innovative knowledge and training by healthcare teams, and the recognition of individual differences in the delivery of pain management services [5,6,7,8,9].

The complexity of patients’ pain should ideally be addressed through intermediate care units if it persists for long [10,11,12]. The complexity of patients’ pain refers to the multifactorial nature of pain experienced by some patients. Pain can be influenced by various factors, including biological, psychological, and social factors. Patients with complex pain may have multiple underlying conditions that contribute to their pain, and pain management can be challenging [10,11]. For example, a patient with chronic back pain may have underlying psychological factors, such as depression or anxiety, that contribute to their pain experience. In addition, social factors, such as employment status or family support, can also influence their pain experience [11,12,13]. In such cases, pain management may require a multidisciplinary approach that addresses the underlying factors contributing to the pain. Intermediate care units, as mentioned in the initial statement, can provide specialized care for patients with complex pain. These units typically provide a higher level of care than standard hospital units and are staffed by healthcare professionals with expertise in pain management. Intermediate care units can provide comprehensive pain management plans that address the multifactorial nature of pain and incorporate a range of interventions, including pharmacological and non-pharmacological approaches. In comprehensive pain management, a multidisciplinary team of healthcare professionals usually collaborate to develop an individualized plan for patients, considering each patient’s unique situation with the aim of reducing pain and improving wellbeing, function, and quality of life of the patients; therefore, the complexity of patients’ pain highlights the need for a holistic approach to pain management that considers the multiple factors that contribute to pain. Intermediate care units can provide specialized care for patients with complex pain and ensure that they receive comprehensive pain management that addresses their individual needs [10,11,12]. The implementation of the pain management programmes and ongoing training can be a major factor influencing healthcare perceptions of pain management [4,8,13].

As a member of multidisciplinary teams, nurses can play a vital role in educating patients about pain management and helping them to understand the importance of adhering to their prescribed treatment plan. This includes not only providing medication but also using non-pharmacological interventions, such as positioning, massage, and relaxation techniques, to help manage pain [10,11,12,13,14]. Previous studies have identified the barriers to adequate pain management. Nurses are obligated to be knowledgeable about pain assessment and management in order to help patients manage their discomfort. In addition, it has been demonstrated that some nurses focus primarily on communication between patients and nurses/physicians, rather than direct communication between clients and caregivers. Conversely, it was found that nurses did not focus on direct input from patients and families but, instead, relied on their observational and quantitative measurement tools to assess pain and manage it accordingly [11,12]. Nursing strategies based on Orem’s self-care theory emphasize the importance of individuals taking an active role in managing their own health and well-being [6]. In the context of pain management, this means that nurses must support and empower patients to participate in their own pain management by providing education, resources, and support that promote self-care and self-management. This nursing model can be applied to pain management by encouraging patients to take an active role in their pain management by engaging in self-care activities [6]. These include exercise, stress relief and other complementary therapies. Nurses can provide training and resources for patients to promote these self-care activities and empower them to manage pain [4,5,6,7,8]. 

According to our studies in Thailand [11,12], post-operative pain is assessed with vital signs regularly in specific documentation sheets, during the assessment of patients in pain; by including self-reported patient pain in their assessment, nurses would enhance the quality of pain assessment while empowering patients to manage their pain. Nurses in Thailand use pharmacology and non-pharmacologic strategies to manage pain [11,12]. They are employed at various levels novice, advanced, beginner, competent, proficient, and expert by qualified practitioners [14]. They work on pain management activities in many contexts other than specialised surgical, palliative, oncology, or intensive care. In Thailand, surgical nurses provide acute and chronic pain management services for patients in different contexts and situations. Nursing strategies involve extensive knowledge and experience of the institutional process, which can affect pain management. Increasing this knowledge can lead to a better comprehension of the service process.

### Study Purpose

This study aimed to describe surgical nurses’ perceptions of strategies for enhancing their pain management proficiency.

## 2. Materials and Methods

### 2.1. Study Design

The data in this study were collected from open-ended questions that were distributed in a larger three-round Delphi project [8]. Data consisting of answers to three open-ended questions from two sequential questionnaires were collected from forty participants (see Table 1).

A qualitative design was used to explore the strategies used by surgical nurses to enhance pain management proficiency. Data were collected using three open-ended questions. The instructions were as follows: (1) Identify the elements that make a practical and effective nursing strategy for managing pain. (2) What do you think are the most important components of a nursing strategy that improves pain management? (3) Name at least three things that would help the patient with pain. The questions were generated based on a review of policy documents on the core components of pain management programmes for surgical nurses. This review identified three components beneficial for nurses in learning or improving their pain management strategies. The first version of the questionnaire was piloted among nurse teachers to determine whether the questions could be understood well and answered and were adjusted accordingly. 

### 2.2. Setting and Informants

The inclusion criteria were as follows: (1) having at least a Bachelor of Science degree in nursing, (2) working as a surgical nurse, and (3) having at least six years of nursing experience in caring for patients with pain. Forty participants participated in this study. They all worked full-time and were aged between 30 and 68 years, with an average age of 44.75 years. Most participants were women (*n* = 34), and all participants possessed a Master of Science degree in Nursing (MScN). The demographic characteristics of the participants are shown in Table 1.

### 2.3. Data Analysis

We followed Braun and Clarke’s six-phase approach to undertaking general purpose thematic analysis [15]. The Microsoft Excel worksheet format used consolidated criteria for reporting qualitative research (COREQ) management software. The phases included becoming familiar with reading and rereading data, identifying initial code generation, researching, and reviewing themes, identifying naming themes, and reporting data trends. 

The process involved progression from the description in which the data were organised and summarised to a data interpretation process. The first and second authors jointly performed all phases of the analysis and confirmed initial findings with the third and fourth authors. Furthermore, the entire research group repeatedly discussed the emerging analysis.

The rigour of the analysis was considered throughout the entire process. The first step in the analysis was to review the data through several readings to obtain a comprehensive understanding of the content. All data were analysed to determine how they were related to the purpose of the study. It was necessary to identify excerpts that could be used as strategies by surgical nurses in pain management. The second phase involved code creation. The data were systematically reread to organise and generate the initial codes related to the content without losing meaning. Consequently, data with similar content were colour-marked. In the third stage, an initial attempt was made to create a potential theme. The 402 coded data were sorted into groups with similar content, yielding the initial themes. The data were finally categorised into three thematic sections: (i) the elements that make a practical and effective nursing strategy for managing pain (67 responses), (ii) the most important components of a nursing strategy that improves pain management (120 responses), and (iii) elements that would assist the patient in pain management (163 responses) and miscellaneous suggestions (52 responses). The fifth phase focused on identifying and naming themes. Three themes emerged from the analysis; they are presented in the findings section.

### 2.4. Ethical Considerations

Ethical guidelines were followed in this qualitative study [15,16,17]. According to Swedish and Thai national legislation, no official ethics committee permit was required. The research was also not intended to have any physical or psychological influence on the participants [16,17,18,19,20,21]. Informed consent was obtained from all study participants after they were given oral and written information. The participants were informed of the research purpose and methods in person by the experts involved in the study. All the participants could discontinue at any time, and all the data were processed securely so that no unauthorised person would have access to the material. In order to maintain the confidentiality of the participants, pseudonyms were assigned, and age ranges were provided instead of exact ages, whenever a quotation was required.

## 3. Results

**Figure 1:**
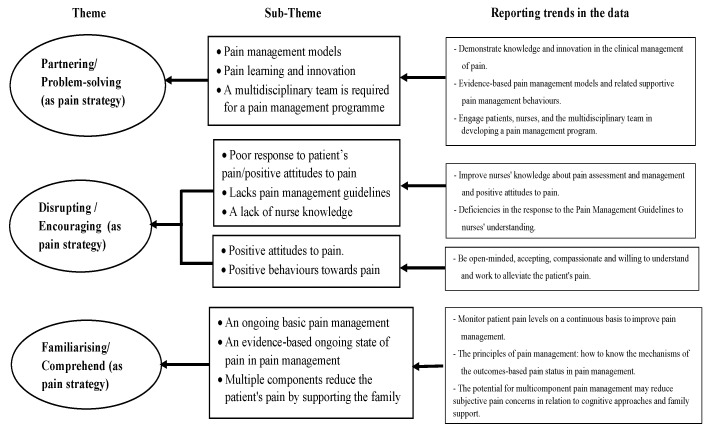
Themes and subthemes that emerged from data analysis describing surgical nurse strategies that enhance their pain management proficiency.

Surgical nurses’ perceptions of the strategies for enhancing pain management proficiency are presented in three themes, including partnering, disrupting, and familiarising oneself with concerns regarding pain management skills (Figure 1). Such strategies in acute and chronic pain management services include solving patients’ problems and encouraging and improving pain management strategies for addressing health challenges within organisations. The themes presented in the results encompassed improvements in pain management and nursing competencies. Advanced healthcare technologies are currently being applied to pain management.

### 3.1. Partnering/Problem-Solving

A nurse’s interpersonal response to pain management encompasses three elements of the healthcare context: engaging patients, relatives, and the multidisciplinary care team. Special skills are needed to identify the learning and innovation needs for a successful pain management. Improving nursing competencies demands knowledge and innovation in clinical pain management. One participant emphasised this through a direct comparison with professionals as follows:

*Knowledge of evidence-based techniques for managing pain. Nursing skills in developing models for managing pain. The integration of pain management concepts is applied alongside other theoretical concepts and the development of innovations in pain management. [Female: Nana, age range: 40–49]*

A multidisciplinary team is required for a pain management programme: All participants acknowledged that patients, nurses, and the multidisciplinary team should be involved in developing a pain management programme. In addition, all participants were aware that the pain management programme included evidence-based pain assessment and management that could be used to treat patients and their families. All participants recognised the importance of nursing strategies and caring for patients in a dignified manner.

*Pain management education programmes present a challenge to nurses. Successful pain management educational programmes require multidisciplinary collaboration. [Female: Wila, age range: ≥60]*

### 3.2. Disrupting/Encouraging

The term ‘disrupting’ is linked to delays in the nursing strategies of a surgical nurse in treating a patient’s pain; examples include poor response to the patient’s pain, lack of pain management guidelines, and lack of nursing knowledge. The response to pain management guidelines has gaps that nurses must understand due to their pain management skills. The participants described the following elements:

*Lack of nursing knowledge is a major hindrance to good pain management practices. Therefore, better-educated nurses are a step in the right direction toward an optimal programme of pain management practices. Nursing education should focus on the development of specific strategies to effectively teach nursing students about pain management as well as the integration of pain management content as a major component in the curriculum for developing effective pain management programmes. [Female: Sani, age range: 40–49]*

In terms of strength in pain management, the majority of the participants reported a nursing strategy using the term ‘encouraging’. Encouragement, including positive attitudes and behaviours toward pain, has been identified as a pain management strategy to support nurses. Nurses should also be encouraged to improve their knowledge of assessing and managing pain and their positive attitudes towards pain. Most participants also expressed open-mindedness, acceptance, compassion, and a willingness to understand and work to alleviate the patient’s pain.

*Things like (1) mindset or attitude towards managing pain in nurses, (2) managing pain as a lifestyle for nursing practice because it is important to care for patients who are suffering, and (3) ongoing improvements in pain management quality, such as participation in pain management training. [Female: Jine, age range: ≥60]*

### 3.3. Familiarising/Comprehending

All participants described the strategies of surgical nurses who used in-person care as a part of pain management. It was identified that nurse strategies typically provided surgical nurses with a familiarity with the concept of ongoing basic pain management.

Nurses can improve care delivery by monitoring patients’ pain levels on an ongoing basis to improve pain management. Additional multicomponent training reduces patients’ pain by supporting the family, involving a continuous, evidence-based state of pain in pain management. All recognised importance and examples include the principles of pain management: knowing the mechanisms of the outcome-based state of pain in pain management.

*Nurses in pain management roles are continuously assessed for performance. Identify knowledge and nursing practice indicators of pain management. Structure of knowledge development system and competencies according to performance. Develop staff development plans based on tasks and training requirements; develop incentives for learning and work development, such as quality competitions and university presentations in foreign countries. [Male:Pate, age range: 40–49]*

Participants emphasised the benefits of developing nursing strategies to heal surgical patients through pain management. They described the benefits of collaborative approaches to nursing and highlighted the importance of involving patients’ families in their care. It notes that multiple components can be used to reduce a patient’s pain and that sustaining their family is an important aspect of this.

The statement also emphasizes the role of teamwork in achieving successful pain management outcomes. Participants identified teamwork as a benefit, as it enables healthcare professionals to work together to develop and implement a comprehensive pain management plan that incorporates multiple interventions. The statement goes on to suggest that nurses may have subjective concerns about cognitive approaches and family support; however, by addressing these concerns and implementing effective pain management strategies, nurses can better address challenging situations and provide better care to their patients.

In summary, the statement underscores the importance of a collaborative approach to nursing, particularly in the context of pain management. It emphasizes the need for healthcare professionals to work together to develop and implement comprehensive pain management plans that take into account multiple components and involve patients’ families. Finally, the statement encourages nurses to address their concerns about cognitive approaches and family support in order to improve their ability to manage pain effectively. Additionally, participants described excellent collaborative approaches to nursing as beneficial for both patients and their families. They often understood that multiple components reduce a patient’s pain by sustaining their family. Most participants identified teamwork as a benefit, and a good team enhanced the potential for multicomponent pain management. Nurses can reduce their subjective concerns regarding cognitive approaches and family support, thereby, enabling them to address challenging situations.

## 4. Discussion

This study provides insight into the experiences of surgical nurses, shedding light on their perceptions of the nursing strategies that they employ to enhance pain management proficiency. Notably, the participants were not well-versed in the principles of sustainability. Our knowledge has allowed us to address challenges with respect to the concepts of partnering, disrupting, and familiarising. Nurses are problem-solving partners in pain management. Reducing delayed treatment spaces is disruptive because it helps patients and families understand the pain management infrastructure. 

Our results demonstrate the complexity of implementing nursing strategies to improve nurses’ pain management skills. Nurses taking care of patients with pain recognised that this was part of their professional role, as mentioned in previous studies [5,6,7,8,9,12]. Nurses reported partnering with a ‘multidisciplinary team’ for problem-solving while caring for patients in recovery surgery. Nurses enhance their competencies through training in pain management practices [3,4]. It is important to consider the following five issues regarding nurses’ responses to patient’s pain: (1) The importance of pain assessment: Nurses should assess their patients’ pain regularly and use appropriate tools to evaluate the intensity and quality of the pain. This can help to guide pain management interventions and ensure that patients receive adequate relief. (2) Communication with patients: Nurses should communicate effectively with their patients about their pain and encourage them to report any discomfort or changes in their pain experience. This can help to ensure that patients feel heard and understood and that their pain is managed appropriately. (3) Use of multimodal pain management: Nurses should be trained in using multimodal pain management, which encompasses a comprehensive approach to pain management and necessitates a collaborative effort from a multidisciplinary team to achieve optimal outcomes for patients. This education can include the use of pain medication, relaxation techniques, and physical therapy, for example. Nurses play a critical role in coordinating and implementing multimodal pain management strategies, monitoring and assessing patient response, and providing emotional and psychological support to patients. (4) Collaboration with other healthcare professionals: As mentioned in the initial statement, nurses can partner with a multidisciplinary team to problem-solve and manage patients’ pain more effectively. Collaboration with other healthcare professionals, including physicians, pharmacists, and physical therapists, can help to ensure that patients receive comprehensive pain management. (5) Compassionate care: Nurses should recognise that pain is a complex and multifaceted experience that requires a holistic approach to care that is both effective and compassionate [6,7]. This recognition can help to build trust between patients and nurses and improve patients’ overall experience of care [5,6,7,8,9,12]. Overall, nurses’ responses to patients’ pain should be guided by regular pain assessments, effective communication, multimodal pain management, collaboration with other healthcare professionals, and compassionate care. By enhancing their competencies through training in pain management practices, nurses can better support their patients’ pain management needs and improve their overall quality of care.

It is also important to consider nurses’ attitudes towards patient care in their education. Nurses’ attitudes towards pain can have a significant impact on how they manage their patients’ pain. Negative attitudes or misconceptions can lead to inadequate pain management and negatively affect patients’ quality of life [17,22]. Prevalent attitudes among nurses: Research has shown that some nurses hold beliefs that pain is inevitable or that patients exaggerate their pain. These attitudes can prevent nurses from taking pain seriously and providing appropriate pain relief. The need for training and education: To address these negative attitudes, it is important to provide training and education for nurses on the appropriate management of pain. This can include education on pain assessment, medication administration, and non-pharmacological pain management techniques. The importance of empathy and communication: Nurses should also be trained to communicate effectively with patients about their pain and to show empathy towards their pain experiences [10,11,12]. This can help to build trust between patients and nurses and improve patients’ overall experience of care. The role of interdisciplinary teams: Effective pain management often requires input from a range of healthcare professionals, including physicians, pharmacists, and physical therapists. Nurses should be encouraged to work collaboratively with these professionals to ensure that patients receive comprehensive pain management. Thus, addressing negative attitudes towards pain among nurses requires a multifaceted approach that involves education, communication, and collaboration with other healthcare professionals [17,22].

One of the challenges that expert nurses reflected on in this study was their lack of communication skills. A nursing strategy to encourage surgical nurses involves engaging with patients, family members, and multidisciplinary care teams. A study by Chatchumni, Namvongprom, Eriksson, and Mazaheri [23] reported on ‘Responding to and addressing patients’ postoperative pain system model’, along with the overlap of pain management systems. The study shows that clinical nurses with good communication skills, can establish a relationship of empathy and trust, and enable a holistic assessment of value judgements in pain management [22,23,24,25]. 

These results indicate a transfer of knowledge to the practice of multicomponent training, which reduces patient pain. Additionally, nurse strategies should focus on family support, which involves an ongoing evidence-based state of pain management. It should also be noted that knowledge-based training is effective in improving postoperative pain practices. Further research is important for developing a pain management programme with appropriate attitudes and symptoms and sharing experiences across clinical and non-clinical nursing [7,12]. Our results suggest that in regard to nurse strategies in pain management, it is important to consider the following points: (1) Family support: As mentioned in the initial statement, nurses can focus on family support as a strategy to manage pain. This can involve educating family members about pain management techniques, providing emotional support to both the patient and family members, and involving family members in the care plan. (2) Evidence-based practice: Nurses should base their pain management strategies on evidence-based practice, which involves using the best available evidence to guide clinical decision-making. This can involve staying up-to-date with the latest research in pain management and using established pain management protocols. (3) Individualized care: Pain management strategies should be individualized to each patient’s unique pain experience and care needs. This can involve tailoring pain medication dosages to each patient, using non-pharmacological interventions that are most effective for each patient, and adjusting pain management plans as needed. (4) Interdisciplinary collaboration: Effective pain management often requires collaboration with other healthcare professionals, including physicians, physical therapists, and pharmacists. Nurses should be encouraged to work collaboratively with these professionals to ensure that patients receive comprehensive pain management. (5) Patient education: Nurses should educate their patients about pain management techniques, including the use of pain medication, relaxation techniques, and physical therapy. Patients should also be encouraged to report any changes in their pain experience to their healthcare provider and to ask questions about their pain management plan. 

Overall, our study findings suggest nurse strategies in pain management that should focus on family support, evidence-based practice, individualized care, interdisciplinary collaboration, and patient education. By incorporating these strategies into their practice, nurses can better support their patients’ pain management needs and improve their overall quality of care. 

### Strengths and Limitations

Necessary measures were taken to ensure trustworthiness of the study. Trustworthiness in research refers to the credibility, transferability, dependability, and confirmability of the research findings [26]. This study included qualitative descriptions of the diverse experiences of surgical nurses in pain management using three open-ended questions. All four authors contributed to data analysis and the construction of the themes. The authors discussed the different interpretation of the data and thematisation of data to reach a consensus on the results. The authors have extensive experience as researchers and have conducted qualitative analysis previously. Our efforts were to ensure that the data analysis process was rigorous and transparent, and that the findings were accurate and meaningful. Member check could have been a fruitful strategy to enhance the study rigor but was not performed in this study considering the hectic workload of the study participants. 

We believe that the qualitative survey approach was a suitable choice for our study, where researchers seek to understand the subjective experiences, perspectives, and meanings of the participants’ experience [26]. We might have been able to collect richer data by interviewing the participants instead of asking them to write down their perceptions; however, considering the participants’ heavy workload as surgical nurses, booking interview sessions with them could have been a challenge. By choosing written open-ended questions as data collection method, we were able to include 40 surgical nurses and analyse their perceptions of strategies for enhancing their pain management proficiency. The participants were able to answer the questions when they had time and concentration. 

This study is presumed to be the first to examine the experiences of surgical nurses in pain management in Thailand; however, only 40 surgical nurses with six or more years of nursing experience in treating patients with pain were interviewed. Therefore, the results could not be generalized considering the study sampling method and qualitative approach of the study; however, the results are transferable in similar contexts. It needs to be noted that most of our study participants had at least a master’s degree in nursing science and at least six years of experience as clinical nurse, which may have impacted the results; therefore, the results should be interpreted and implemented carefully. 

## 5. Conclusions

Participants identified surgical nursing strategies for managing patients’ pain as a fundamental component of their role; however, this study identified challenges related to partnership, disruption, and familiarisation with pain management. It would be beneficial to increase the knowledge about nursing strategies and support sustainable decision-making in patients’ pain management. Multidisciplinary teams play a pivotal role in problem-solving by encouraging patients and families to understand the pain management infrastructure. Specialised training in pain management is essential to support the outcome-based state of pain in pain management, regardless of whether it involves teaching particular nursing skills or enabling open and honest thinking.

Surgical nurses prefer a greater understanding of the sustainability of nursing strategies in pain management but do not connect with each other in their work. With the help of nursing strategies, especially in terms of postoperative recovery time, it is easier to view the surgical area as part of a broader context. Further research and education are needed on acute and chronic pain management strategies for surgical nurses. Recommendations are required to engage patients, family members, and multidisciplinary care teams in other healthcare areas.

## Figures and Tables

**Table 1:** Sociodemographic characteristics of the participants [8].

	*n*	%	Min	Max	Mean	SD
Age			30	68	44.75	8.53
Gender						
Male	6	15.00				
Female	34	85.00				
Total	40	100.00				
Occupation						
University lecturers/researchers	27	67.50				
Clinical nurses						
Semi-ICU	3	7.50				
Surgical ward	3	7.50				
ICU	2	5.00				
Nurse Anaesthetists	2	5.00				
OPD-Medical	1	2.50				
Medical ward	2	5.00				
Total	40	100.00				
Education						
MScN	26	65.00				
Doctor of Philosophy (candidate)	6	15.00				
Doctor of Philosophy (degree-holder)	8	20.00				
Total	40	100.00				
Experience in nursing care (years)			6	45	20.37	7.63
Special education and training in pain management						
Yes	6	15.00				
No	34	85.00				
Total	40	100.00				

## Data Availability

The datasets generated and analyzed during the current study are not publicly available to uphold informed consent and maintain confidentiality but can be obtained from the corresponding author upon reasonable request.
